# Interactive effects of strontium and barium water concentration on otolith incorporation in juvenile flounder *Paralichthys olivaceus*

**DOI:** 10.1371/journal.pone.0218446

**Published:** 2019-06-14

**Authors:** Honglin Tian, Jinhu Liu, Liang Cao, Shuozeng Dou

**Affiliations:** 1 CAS Key Laboratory of Marine Ecology and Environment Sciences, Institute of Oceanology, Center for Ocean Mega-Science, Chinese Academy of Sciences, Qingdao, China; 2 University of Chinese Academy of Sciences, Beijing, China; 3 Laboratory for Marine Ecology and Environmental Science, Qingdao National Laboratory for Marine Science and Technology, Qingdao, China; Department of Agriculture and Water Resources, AUSTRALIA

## Abstract

Although the relationship between the incorporation of an element into otoliths and the concentration of the element in water has been extensively investigated in many fish species, the interactive effects of multiple elements in water on the otolith incorporation of an element are not adequately explored or well understood. In this study, 16 treatments in triplicate using strontium (Sr; 1, 2, 3 and 4 times the ambient baseline, 6.5 mg l^-1^) and barium (Ba; 1, 2, 4 and 6 times the ambient baseline, 40 μg l^-1^) as categorical variables in an orthogonal design were established to evaluate the relative or interactive effects of water elements on otolith elemental incorporation in juvenile flounder *Paralichthys olivaceus* (from 15 to 116 days post hatching). The results revealed that otolith incorporation (Me:Ca_Otolith_) of Sr and Ba were positively dependent on the concentrations of the elements in water (Me:Ca_Water_). Overall, Sr was incorporated into otoliths more efficiently than was Ba, and the partition efficiency (D_Me_) of both elements decreased with increasing water elemental concentrations. Increasing Sr concentrations in water appeared to negatively affect the uptake of Ba into otoliths rather than facilitate it, as previously reported in fish reared in freshwater and brackish water, or showed no effect on fish in seawater. Conversely, the Ba concentration in water did not influence the otolith uptake of Sr, which agrees with the findings for other fish species. When applying otolith microchemistry to fish ecology studies, it is essential to cautiously address the interactive effects of multiple elements in the environment on otolith elemental incorporation.

## Introduction

Fish otoliths are chemically composed of calcium (Ca) and a variety of trace elements, which are primarily incorporated from ambient water throughout their life. Once crystallized and deposited in the inner ears of fish, otoliths are physiologically stable because of metabolic inertness [1, 2]. It is commonly assumed that trace elements deposited in otoliths during crystallization cannot be decomposed or reabsorbed, thus making otolith microchemistry a useful natural logger for recording the environmental history of fish [2–4]. Accordingly, otolith microchemistry analysis is a useful tool to address a variety of issues in fishery ecology. For example, elemental fingerprinting can be used to reconstruct migratory histories [5], identify natal origins or nursery habitats [6], assess movement patterns [7, 8] and discriminate stocks [9, 10].

Otolith chemistry has been reported as a reliable indicator of environmental histories for many species [11, 12], although the rates of elemental incorporation have been shown species-specific dynamics [4, 13]. During the process of elemental incorporation, trace elements are likely to substitute for Ca^2+^ within the calcium carbonate (CaCO_3_) matrix of otoliths, which is usually related to the ambient elemental concentration [2, 4, 14, 15]. Since otoliths are not in direct contact with the environment due to physiological barriers, elements must cross the gills or tissue cells, and then the ear membranes via blood circulation before they are finally incorporated into otoliths [1, 2]. Thus, all processes affecting ion transport, binding and availability for incorporation have the potential to influence otolith elemental incorporation [4, 12]. In fact, both environmental (*e*.*g*., temperature and salinity) and physiological factors (*e*.*g*., ontogeny, growth and reproduction) may alter otolith microchemistry directly, as well as indirectly, by altering internal metabolic and physiological processes that influence elemental incorporation [2, 4, 12, 16]. To date, several studies have validated the positive or negative effects of temperature and salinity on the incorporation of trace elements in the otoliths of some fish species [17–21], although such effects were not significant in other species [18, 22].

During the formation and growth of otoliths, multiple trace elements with distinct chemical properties may be simultaneously incorporated into otoliths from ambient water. Since elemental ions may compete for space in lattice sites in the otolith matrix during crystallization, elements can potentially interact during the incorporation of a specific element in the otolith. Previous studies have commonly investigated the incorporation of an element independently and rarely considered the potential competition or facilitation by other elements. Two studies addressed this issue and demonstrated that increasing Sr concentrations in water facilitated the uptake of Ba in the otoliths of black bream *Acanthopagrus butcheri* juveniles reared in brackish water [15] and freshwater yellow perch *Perca flavescens* juveniles [23]. However, no such “elemental effect” has been reported in marine fish. Currently, we have insufficient knowledge to clarify if or how multiple elements in the environment interact with the uptake of elements in otoliths. More research is needed to test and assess how the interaction of multiple ambient elements may influence otolith elemental incorporation.

Flounder *Paralichthys olivaceus* is an important commercial fish that is widely distributed in the coastal waters of East Asia. Flounder spawns from April to June in shallow nearshore waters. After hatching, larvae are transported to coastal waters, where they finish metamorphosis and then settle. The newly settled juveniles tend to move to offshore nursery areas to forage and grow until they migrate to deep overwintering areas. In nature, all these physiological and ecological traits represent dramatic shifts in biological and environmental histories, which could eventually affect otolith microchemistry. Additionally, flounder has been bred for aquaculture and stock enhancement in East Asian countries for many years. Flounder obtain relatively stable and high survivorship once the larvae survive a “critical period” of mortality around first feeding [24]. Furthermore, growth depensation (*i*.*e*., individual growth variation enlarged by social interaction due to size hierarchies) occurs due to aggressive attacking and cannibalism in the early juveniles [25]. These advantages favour flounder as an ideal candidate fish for an experimental study to identify the factors that regulate otolith microchemistry, especially when the experiments are designed to start from the embryonic or early larval stage.

We used flounder as an experimental fish and conducted a laboratory experiment to investigate how elemental concentration might affect otolith elemental incorporation during early life stages. In this experiment, flounder were reared from the early larval stage to juveniles for over 10 weeks. The target elements that were examined were Sr and Ba, which are the two most investigated elements because of their active incorporation into otoliths. The current study mainly tested if and how the otolith incorporation of the two elements was correlated to elemental concentration and elemental interaction.

## Materials and methods

### Egg incubation and rearing of larvae

Fertilized flounder eggs were provided by the Shunyuan Fishery Station at Rizhao, Shandong Province. The eggs were acquired from the same broodstock to minimize potential genetic variability among individuals. Approximately 50,000 viable eggs were transferred into a 1000 l concrete pond that was filled with filtered seawater. The water temperature was 17.4±0.5°C throughout egg incubation. The light regime corresponded to the natural light at the laboratory (13 L at 0600–1900 h and 11 D at 1900–0600 h). The eggs started to hatch at *~* 40 h after fertilization (haf). At 60 haf, when hatching was supposed to finish, the hatching rate was estimated to be *c*. 92%.

The larvae started feeding on 3 days posthatching (dph). Following routine rearing practice and feeding management [24, 25], newly hatched larvae were provided the rotifer *Brachionus plicatilis* as food twice per day (at 0800 and 1300 h) until they were used for the experiment. Rotifers were cultured at 21–23°C and fed *Chlorella sp*. Daily water changes via flowing water began on 3 dph, increasing from 30% to 150% of the total water volume with the progression of larval development. Other rearing conditions (seawater, temperature and light conditions) were similar to those used for egg incubation. The incubation and rearing pond were cleaned twice a day by siphoning, and all dead larvae were counted and removed.

At 15 dph, when larvae were assumed to have survived the “critical period” of mortality and were sufficiently developed for experimental handing, they were used for the experiment.

### Experimental design

All experimental tanks were 50 l transparent polypropylene tanks containing 40 l filtered seawater and were provided with mild aeration under a light regime of 13L:11D. The 15 dph larvae (7.9 mm in total length, L_T_) were randomly selected from the rearing pond with beakers, visually counted and stocked in experimental tanks (50 larvae per tank) that were randomly assigned locations, treatments and replications. The water salinity and temperature in the tanks were monitored twice a day (at 0800 and 1400 h).

**Fig 1:**
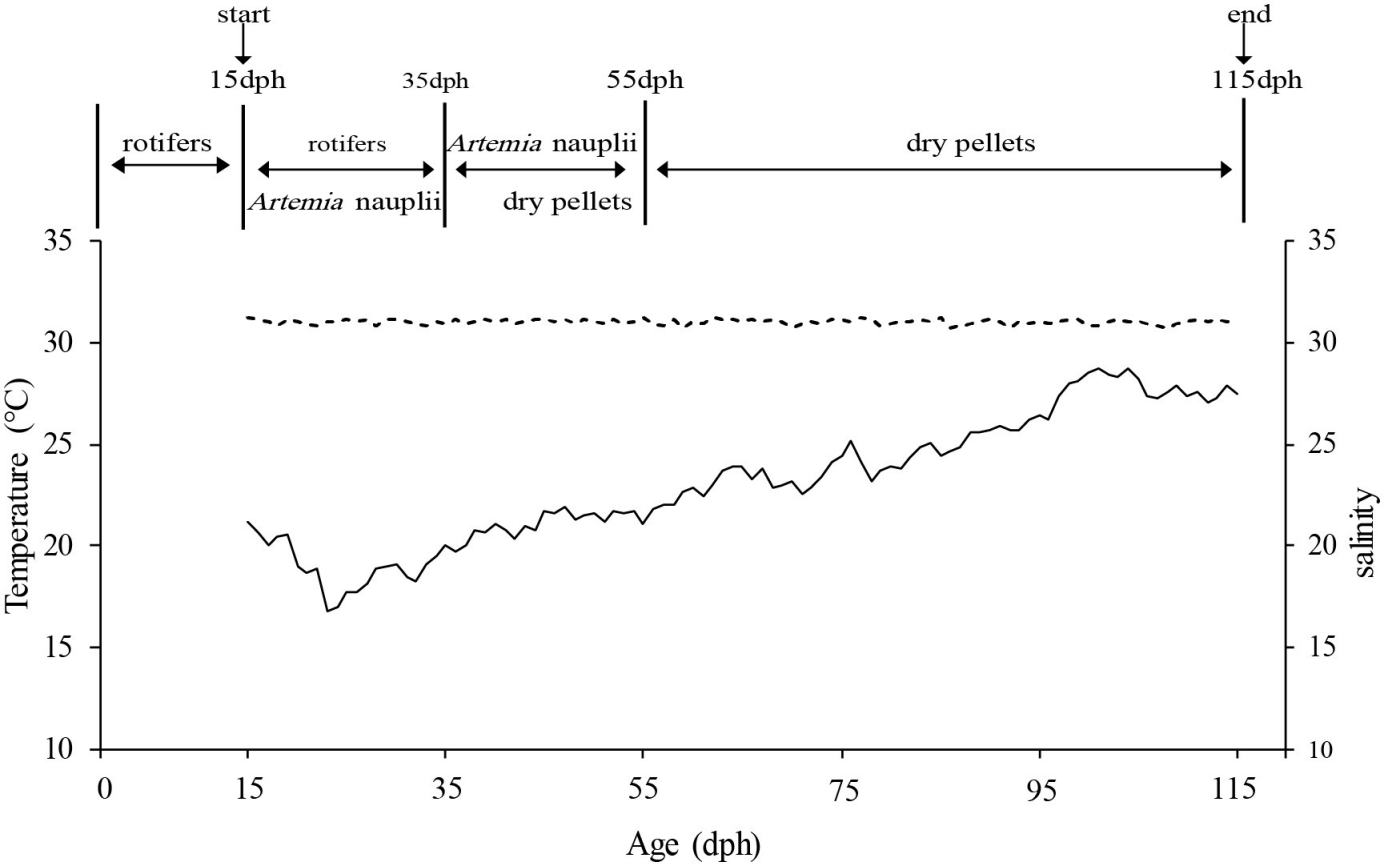
Average daily water temperature, salinity and feeding management across treatments over the course of the experiment. Solid line, water temperature; dotted line, water salinity.

Following our previous practice for rearing flounder larvae and juveniles [24, 25], the rearing management changed with the development of the flounder over the course of the experiment (Fig 1). During the larval stage (15–35 dph), daily water changes of *c*. 80% were maintained, and the larvae were fed rotifers and *Artemia* nauplii after each water change. Starting at 36 dph, the newly settled juveniles were fed *Artemia* nauplii and the commercial dry feed pellets and water were completely changed every day until 56 dph. From then on, fish were weaned onto dry pellets, and the water was completely changed twice a day until the end of the experiment. Water changes were performed by replacing the water of the tanks with filtered natural seawater with desirable elemental concentrations corresponding to the requirements of each treatment. By changing the water in this way, consistent elemental concentrations in the water in the tanks for each treatment could be relatively well controlled and manipulated over the course of the experiment. No prey was offered to the fish during the dark period because they do not feed in the dark. All these rearing conditions are assumed to be appropriate for normal development, survival and growth of the flounder larvae and juveniles [24, 25]. The flounder showed aggressive attacking or cannibalistic behavior, which caused mortality during the experiment. When fish started to show severely broken fins, sluggish movement and stopped feeding, they were considered dying or dead and were immediately removed from the tanks to be euthanized and preserved in liquid nitrogen. The tanks were checked for dying fish every 6 h. Fish mortality ranged from 14% to 25% across treatments (averaging 9 dead individuals per tank) throughout the experiment.

In the experiment, four water elemental concentrations were established for Sr (1, 2, 3 and 4 times the ambient elemental concentration, 6.5 mg l^-1^) and Ba (1, 2, 4 and 6 times the ambient elemental concentration, 40 μg l^-1^). A total of 16 treatments in triplicate, using Sr and Ba as categorical variables in the orthogonal design, were established to investigate the relative or interactive effects of water elemental concentrations on otolith elemental incorporation. Water elemental concentrations were manipulated by the addition of appropriate amounts of standard solutions of SrCl_2_·6H_2_O and BaCl_2_·2H_2_O in fresh seawater. The experiment was terminated when fish reached 116 dph and last for a total of 100 days.

The test protocol was designed in accordance with the recommendations of the Regulations of the Laboratory Animal-Guidelines for Ethical Review of Animal Welfare (Standardization Administration of China, 2018) and approved by the Committee on the Ethics of Animal Experiments of the Institute of Oceanology, Chinese Academy of Sciences (No. 20180258). The principles of reduction, replacement and refinement were carefully followed when we developed the test. During the test, the flounder larvae were reared under routinely practiced culturing conditions to ensure their welfare. At sampling, larvae were immediately sacrificed in liquid nitrogen to alleviate the possible stress and pain. The number of embryos stocked in the tanks was controlled to satisfy the minimal requirement for this test.

### Water and otolith sampling

Starting on the first day of the experiment, 50 ml water was randomly collected from one tank of each treatment over the course of the experiment at 10-day intervals to measure the elemental concentration in the water. After sampling, the solutions were filtered with 0.45 μm film, acidified with 2% ultrapure HNO_3_ (Merck, Germany), and stored in acid-washed polypropylene bottles at 4°C until chemical analysis.

At the end of the experiment, three fish were randomly selected from each tank (a total of 9 fish per treatment) for elemental measurements in otoliths to investigate the incorporation of Sr or Ba in relation to the elemental concentration in water or elemental interactions. After sampling, sagittal otolith pairs were extracted, cleaned with distilled water, air-dried, and then stored in microcentrifuge tubes. As sagittal otoliths are symmetrical in flounder, the right otolith of each pair was then used for chemical analysis.

### Otolith and water element analysis

Otolith sample preparation and elemental measurements using solution-based inductively coupled plasma mass spectrometry (ICP-MS; Elan DRC II, Perkin Elmer) were conducted following the methods of Higgins et al. [10] and Bailey et al. [26]. Prior to the elemental analysis, otoliths were placed into microcentrifuge tubes and sonicated in ultrapure water (18.2 MΩ-cm) for 5 min in an ultrasonic cleaner to remove tissues adhered to the surfaces. Otoliths were then decontaminated in 3% H_2_O_2_ solution for 10 min to eliminate the remaining superficial contaminants. Thereafter, otoliths were rinsed twice with ultrapure water to remove H_2_O_2_ from the surface and dried in a laminar flow fume hood. The dried otoliths were weighed using an analytical balance (Discovery DV215CD, 0.01 mg, Ohaus), rinsed twice with ultrapure water to remove contamination during weighing, and then dried for elemental analysis. All tools for handling otolith samples were acid-washed to reduce contamination during otolith sample preparation.

The right otoliths were fully digested in a specified volume of 10% ultrapure HNO_3_ (Merck) that was proportional to the otolith mass (1 mg/0.5 ml, otolith w/acid v; w, weight, v, volume) to obtain a relatively constant Ca^2+^ concentration across sample solutions so that the potential matrix effects of Ca^2+^ were assumed to be consistent in each sample. Each sample solution was diluted with ultrapure water (1v/4v, sample solution/ultrapure water) to obtain a 2% HNO_3_ matrix solution. When the otolith samples were analyzed on an ICP-MS, instrument drift occurred due to the buildup of Ca^2+^ on the instrument, as well as changes in temperature, nebulizer aspiration rates and plasma, which resulted in low detection limits of the elements. All sample solutions, standards and reference materials were spiked with indium (^115^ In, *c*. 4.5 ppb) as an internal standard to correct for instrument drift as well as potential matrix effects. Elements were quantified by an external calibration method with multi-element standards (Chinese Academy of Geological Sciences) containing the target elements.

During each analysis session, 8 otolith solutions were randomly selected and numbered from all treatments, and these samples were analyzed. The order of otolith analysis was randomized so that the analysis of any treatment could be spread over the entire analysis procedure, thus reducing potential sequence effects. Prior to each analysis session, blank (2% HNO_3_ solution, Merck), multi-element standards and reference materials, all with 2% HNO_3_ solutions as matrixes, were successively analyzed. The certified reference material (NIES-22, National Institute for Environmental Studies, Japan) was analyzed over the course of all analysis sessions to determine the accuracy and precision of the analysis. A recovery rate of 96, 99% and 103% and an average analytical precision of 2.4, 3.1% and 4.5% relative standard deviation (RSD) were achieved for Sr, Ba and Ca, respectively. The blank was analyzed to determine the limits of detection (LODs) for each element, thus validating that the detectable concentrations of Ca, Sr and Ba in otoliths were all well above their LODs.

Water samples were diluted with 2% ultrapure HNO_3_ solution (1v/99v, sample solution/acid solution) and were analyzed for Ca concentration by inductively coupled plasma atomic emission spectrometry (ICP-AES; IRIS Intrepid II XSP, Thermo Electron Corporation, USA) and for Ba and Sr concentrations by ICP-MS (Elan DRC II, Perkin Elmer). The analytical procedures and methods were similar to those used for the otolith elemental measurements. In short, the internal standards and blanks were the same as those used to measure the otolith elemental concentrations. The external standards and reference material were certified standard seawater solutions. During each analysis session, 10 sample solutions randomly selected across treatments were analyzed. The precisions for the Sr, Ba and Ca concentrations in the reference solution were 1.3%, 1.3% and 1.7%, respectively. Meanwhile, the recovery rates were 98% for Sr, 102% for Ba and 96% for Ca.

### Data analysis

All elemental concentration data were standardized to Ca, which was abundant and stable in all treatments, by expressing the elemental concentrations as molar ratios to Ca (Me:Ca_Otolith_). The mean elemental concentrations (mean±SD) of the individuals from each tank were calculated. Elemental error, which was defined as the percentage of the absolute difference of the measured and nominal concentrations to the nominal concentration of each water sample, was used to assess how precisely the nominal water elemental concentrations were reflected throughout the experiment. The partition coefficient (D_Me_), which is the ratio of Me:Ca_Otolith_ to Me:Ca_Water_, was used to assess the efficiency of elemental incorporation into otoliths from ambient water for each individual fish. Me:Ca_water_ was the average of the elemental concentrations measured in a treatment over the course of experiment.

Prior to running ANOVA, the raw data were checked for normality and homogeneity of variance using Kolmogorov–Smirnov and Levene tests, respectively. In this study, a few sets of elemental concentration data did not meet the assumptions for these statistics. Therefore, all elemental concentration data were transformed with ln(x+1). One-way ANOVAs were performed to assess whether rearing conditions (water temperature and salinity) differed among treatments. Two-way ANOVA was used to evaluate the effects of elements in the environment (Me:Ca_Water_) on otolith elemental incorporation (Me:Ca_Otolith_), followed by p*ost hoc* multiple comparisons (SNK test) to determine the significances between treatments. Furthermore, linear regression analyses were performed to examine the correlations between Sr:Ca_Otolith_ and Sr:Ca_Water_ at each Ba concentration or between Ba:Ca_Otolith_ and Ba:Ca_Water_ at each Sr concentration.

The D_Sr_ and Sr:Ca_Water_ were plotted using best fit lines to present the trend of D_Sr_ with Sr:Ca_Water_ at each Ba concentration. Similar plots were created for the trends of D_Ba_ with Ba:Ca_Water_ at each Sr concentration. Differences were considered significant at *P*<0.05. All statistical analyses were performed using SPSS 22.0 for Windows.

## Results

### Experimental conditions and final fish size

The elemental errors of the measured water elemental concentrations across treatments ranged from 0.3% to 9.4% (average 4.9%) for Sr and 1.4% to 9.1% (average 6.0%) for Ba (Table 1). The measured concentrations of both elements in the tanks were overall close to the nominal ones, indicating that they effectively represented the designed gradients of the elemental concentrations. The water salinities in the tanks remained stable throughout the experiments, ranging from 30.7±0.08 to 31.3±0.05 for each treatment (average 31.0±0.12; Fig 1). The water temperatures in the tanks changed with the natural room temperature, increasing from 17.0±0.03 to 27.5±0.13°C (average 23.2±0.04°C) over the course of the experiment (Fig 1). Neither salinity nor water temperature significantly differed between treatments or replicates (one-way ANOVA, *P*>0.50 for all comparisons; Table 1). These results demonstrated that the experimental fish experienced similar water salinities and temperatures throughout the experiment.

**Table 1:** Summary of Sr and Ba water concentrations, elemental errors, final total length (L_T_) and otolith weight (W_O_) for the fish in the experiment.

Treatment		Sr concentration (mg l^-1^)			Ba concentration (μg l^-1^)			Total length (L_T_, mm) and otolith weight (W_O_, mg)			
Sr	Ba	Nominal	Measured	Error (%)	Nominal	Measured	Error (%)	L_T_	CV (%)	W_O_	CV (%)
1x	1x	6.5	6.21 (0.25)	4.5	40	38.16 (2.95)	4.6	97.0 (13.0)	13.6	1.90 (0.49)	24.9
	2x	6.5	6.35 (0.21)	2.3	80	78.90 (3.32)	1.7	98.5 (17.9)	18.2	2.15 (0.69)	32.0
	4x	6.5	6.38 (0.22)	1.9	160	169.15 (7.23)	8.8	100.6 (17.0)	16.9	2.15 (0.68)	31.5
	6x	6.5	6.66 (0.26)	2.5	240	258.12 (8.77)	6.9	99.8 (17.8)	17.9	2.15 (0.70)	32.7
2x	1x	13.0	12.40 (0.54)	4.6	40	39.31 (2.89)	1.4	96.8 (19.2)	19.9	1.99 (0.59)	29.7
	2x	13.0	12.33 (0.63)	5.2	80	76.57 (3.34)	4.3	99.6 (19.3)	19.4	2.15 (0.71)	33.1
	4x	13.0	12.23 (0.60)	5.9	160	168.67 (6.98)	3.0	97.4 (17.7)	18.2	1.97 (0.63)	32.1
	6x	13.0	13.04 (0.91)	0.3	240	257.76 (9.22)	3.3	98.3 (15.4)	15.7	2.04 (0.58)	28.4
3x	1x	19.5	18.63 (1.01)	4.5	40	36.47 (3.19)	5.7	98.8 (16.5)	16.7	2.11 (0.59)	28.2
	2x	19.5	18.42 (0.88)	5.5	80	77.58 (3.15)	5.4	99.2 (14.9)	15.0	2.01 (0.45)	22.1
	4x	19.5	18.53 (0.99)	5.0	160	175.34 (6.16)	9.6	95.6 (16.3)	17.0	1.94 (0.56)	28.9
	6x	19.5	18.10 (1.02)	7.2	240	260.70 (8.93)	8.1	98.8 (16.9)	17.1	2.08 (0.63)	30.3
4x	1x	26.0	24.74 (1.22)	4.9	40	42.75 (3.45)	7.5	99.1 (17.3)	17.4	2.15 (0.62)	29.0
	2x	26.0	23.98 (1.20)	7.8	80	77.38 (3.72)	7.4	97.9 (18.6)	19.0	2.10 (0.67)	31.5
	4x	26.0	24.16 (1.44)	7.1	160	172.90 (5.75)	8.6	96.2 (15.9)	16.5	2.05 (0.61)	29.6
	6x	26.0	23.57 (1.25)	9.4	240	261.79 (10.02)	9.1	100.8 (15.9)	15.8	2.09 (0.60)	28.9

Values in brackets are standard deviations (SD).

The average final fish size (L_T_) and otolith weight (W_O_) of fish in each treatment were 96–101 mm (CV, 13.6–19.9%) and 1.94–2.15 mg (CV, 22.1–33.1%). Neither L_T_ nor W_O_ significantly differed between treatments or replicates (one-way ANOVA, *P*>0.05 for all comparisons; Table 1).

### Water elemental concentrations and elemental incorporation in otoliths

**Fig 2:**
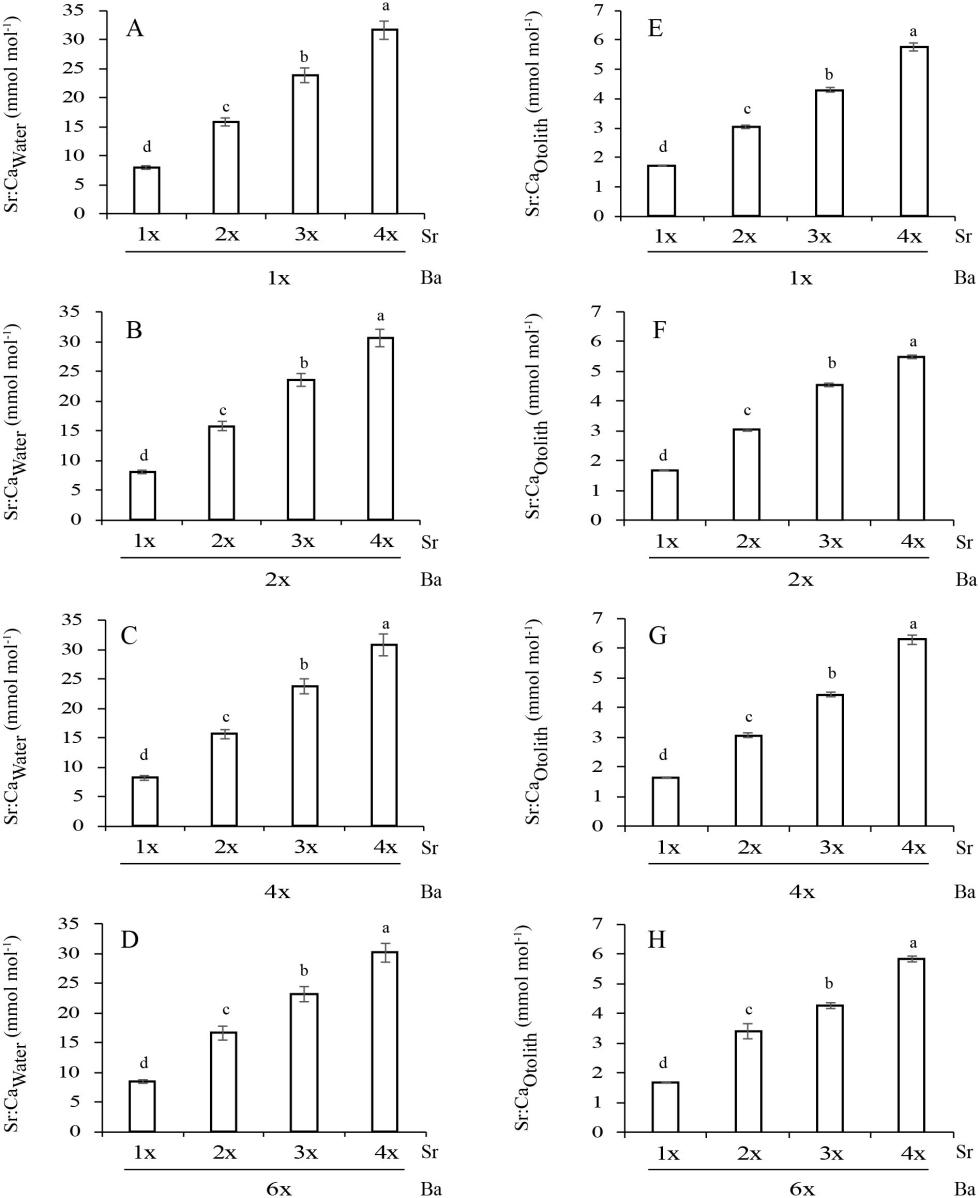
Measured Sr concentrations (mean±SD) in water (Sr:Ca_Water_, A-D) and otoliths (Sr:Ca_Otolith_, E-H) across treatments (two-way ANOVA, *P*<0.05).

**Fig 3:**
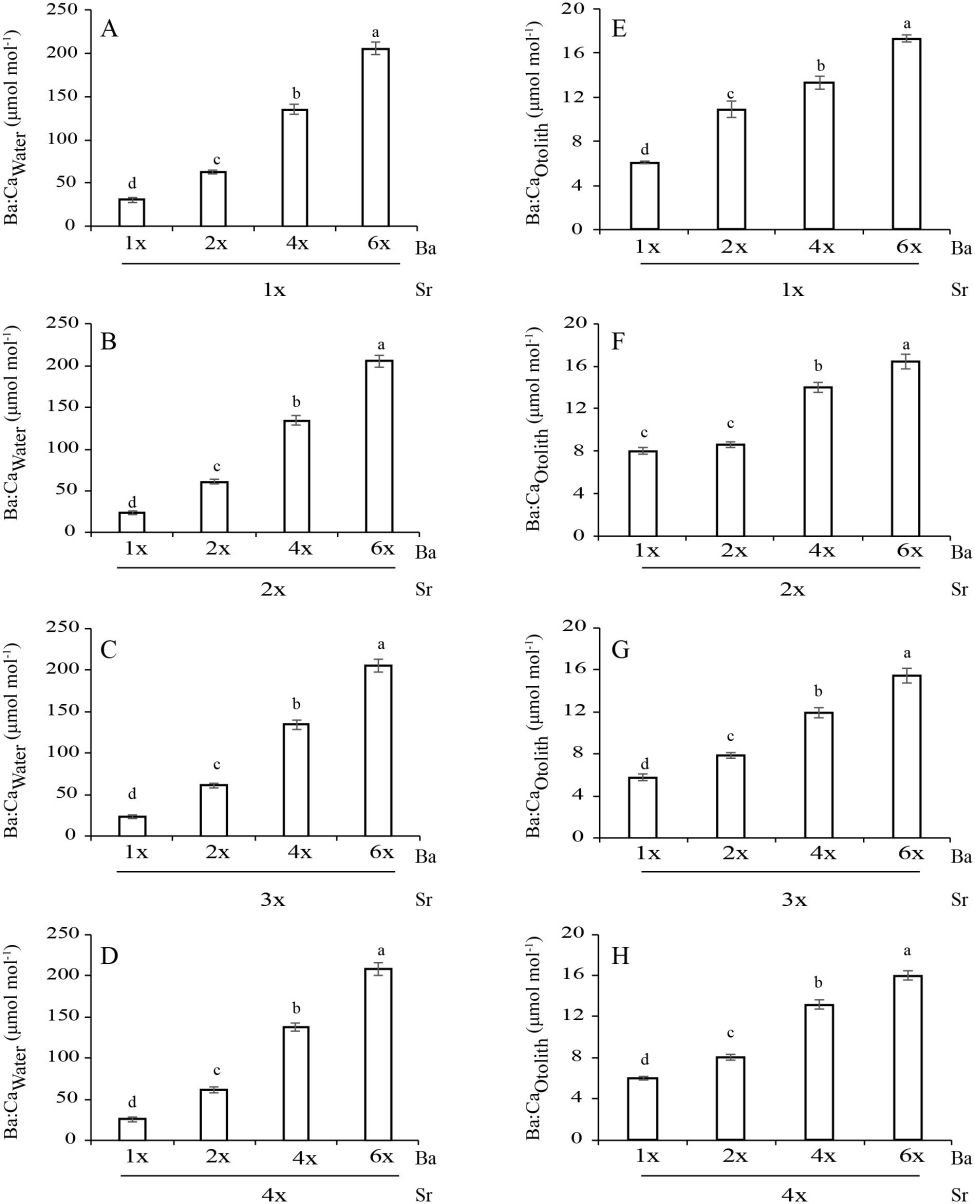
Measured Ba concentrations (mean±SD) in water (Ba:Ca_Water_, A-D) and otoliths (Ba:Ca_Otolith_, E-H) across treatments (two-way ANOVA, P<0.05).

The Sr:Ca_Water_ significantly differed among the Sr concentrations at each Ba concentration (one-way ANOVA, *P*<0.05), although it did not differ between treatments with the same Sr concentration (*P*>0.05; Fig 2A–2D). Similarly, the Ba:Ca_Water_ values significantly differed among the Ba concentrations at each Sr concentration (*P*<0.05), yet they did not differ between treatments of the same Ba concentration (*P*>0.05; Fig 3A–3D). Both Sr:Ca_Water_ and Ba:Ca_Water_ were in accordance with the designed gradients of nominal elemental concentrations.

**Fig 4:**
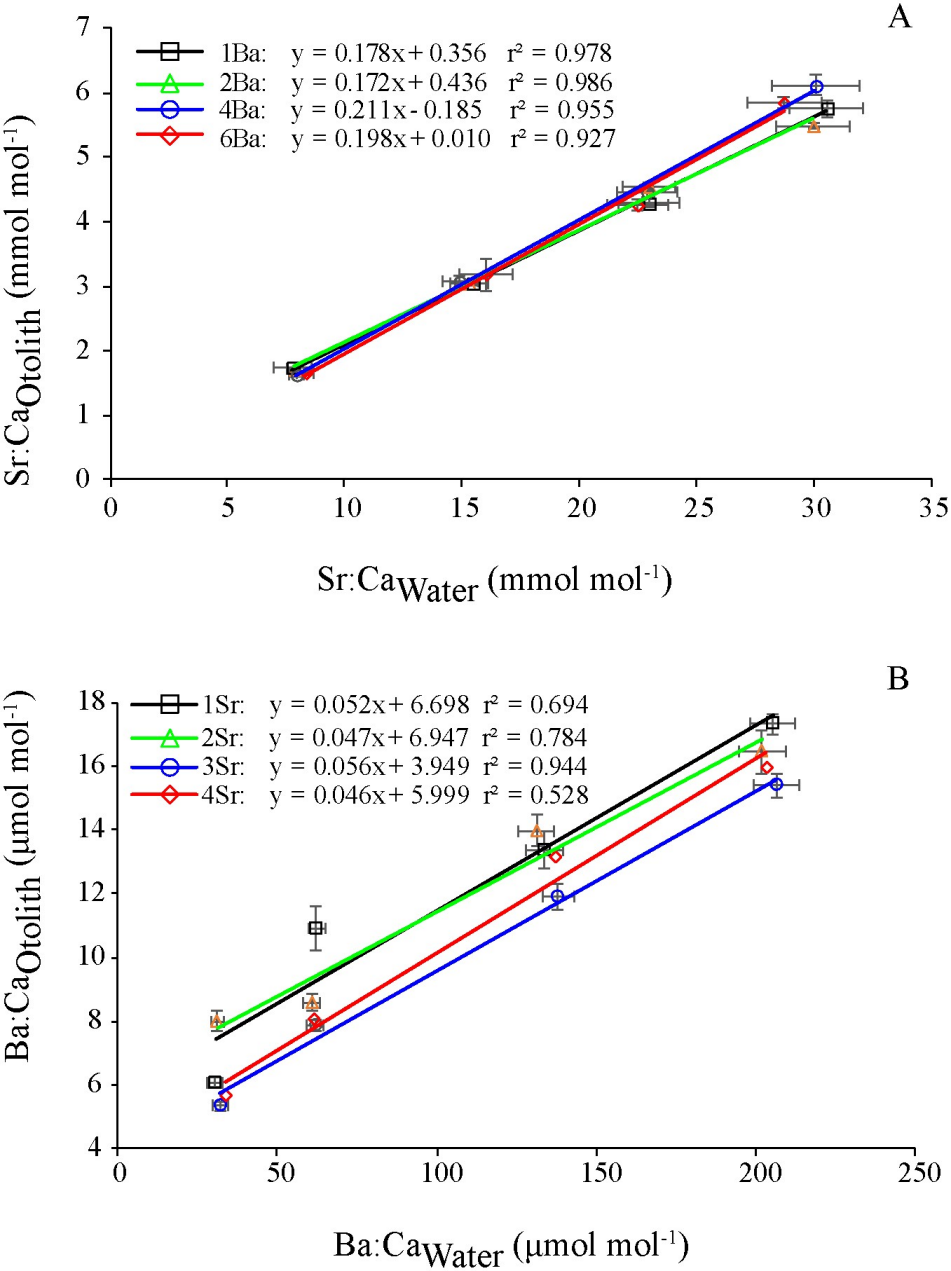
Relationship between Me:Ca_Otolith_ and Me:Ca_Water_ for Sr (A) and Ba (B). Lines were fitted by linear regression analysis at individual elemental concentrations. Data represent the mean values of Me:Ca_Otolith_ or Me:Ca_Water_ for each treatment.

The Sr:Ca_Otolith_ values among the Sr concentrations were 1.73–5.75 (1Ba), 1.70–5.48 (2Ba), 1.62–6.11 (4Ba) and 1.66–5.83 (6Ba) mmol mol^-1^ (Fig 2E–2H). Sr:Ca_Otolith_ was significantly affected by Sr:Ca_Water_, but was not affected by Ba:Ca_Water_ (two-way ANOVA, *P*>0.05; Table 2). Sr:Ca_Water_ and Ba:Ca_Water_ showed interactions with Sr:Ca_Otolith_ (*P*<0.05). At each Ba concentration, Sr:Ca_Otolith_ significantly increased as Sr:Ca_Water_ increased (*P*<0.05, SNK test). At the same Sr concentration, Sr:Ca_Otolith_ did not differ among the Ba concentrations (*P*>0.05). Sr:Ca_Otolith_ and Sr:Ca_Water_ were positively correlated at each Ba concentration (*r*^2^ = 0.92–0.99, *P*<0.05; Fig 4A).

**Table 2:** Results of ANOVA to evaluate the effects of water elemental concentrations (Me:Ca_Water_) on otolith elemental concentrations (Me:Ca_Otolith_) for Sr and Ba.

	Effect	d.f.	MS	F	P
Sr:Ca_Otolith_	Sr:Ca_Water_	3	5.82	1969.10	<0.001
	Ba:Ca_Water_	3	0.001	0.39	0.574
	Interaction	9	0.014	4.84	<0.001
	Tank	2	0.004	1.30	0.277
	Residual	126	0.003		
Ba:Ca_Otolith_	Sr:Ca_Water_	3	0.32	40.05	<0.001
	Ba:Ca_Water_	3	5.42	605.91	<0.001
	Interaction	9	0.08	25.20	<0.001
	Tank	2	0.016	2.053	0.133
	Residual	126	0.008		

The Ba:Ca_Otolith_ values among the Ba concentrations were 6.07–17.32 (1Sr), 8.00–16.44 (2Sr), 5.32–15.39 (3Sr) and 5.61–15.96 (4Sr) μmol mol^-1^ (Fig 3E–3H). Ba:Ca_Otolith_ was significantly affected by both Sr:Ca_Water_ and Ba:Ca_Water_ (*P*<0.05 at both cases; Table 2). Ba:Ca_Water_ and Sr:Ca_Water_ showed significant interactions on Ba:Ca_Otolith_ (*P*<0.05). At each Sr concentration, Ba:Ca_Otolith_ significantly increased as Ba:Ca_Water_ increased (*P*<0.05). Ba:Ca_Otolith_ significantly differed between most Sr concentrations at each Ba concentration (*P*<0.05). A significant but weak negative correlation was detected between Ba:Ca_Otolith_ and Sr:Ca_Water_ at each Ba concentration (*r*^2^ = 0.027–0.120, *P*<0.05). Ba:Ca_Otolith_ and Ba:Ca_Water_ showed a positive linear relationship at each Sr concentration (*r*^2^ = 0.53–0.94, *P*<0.05; Fig 4B).

**Fig 5:**
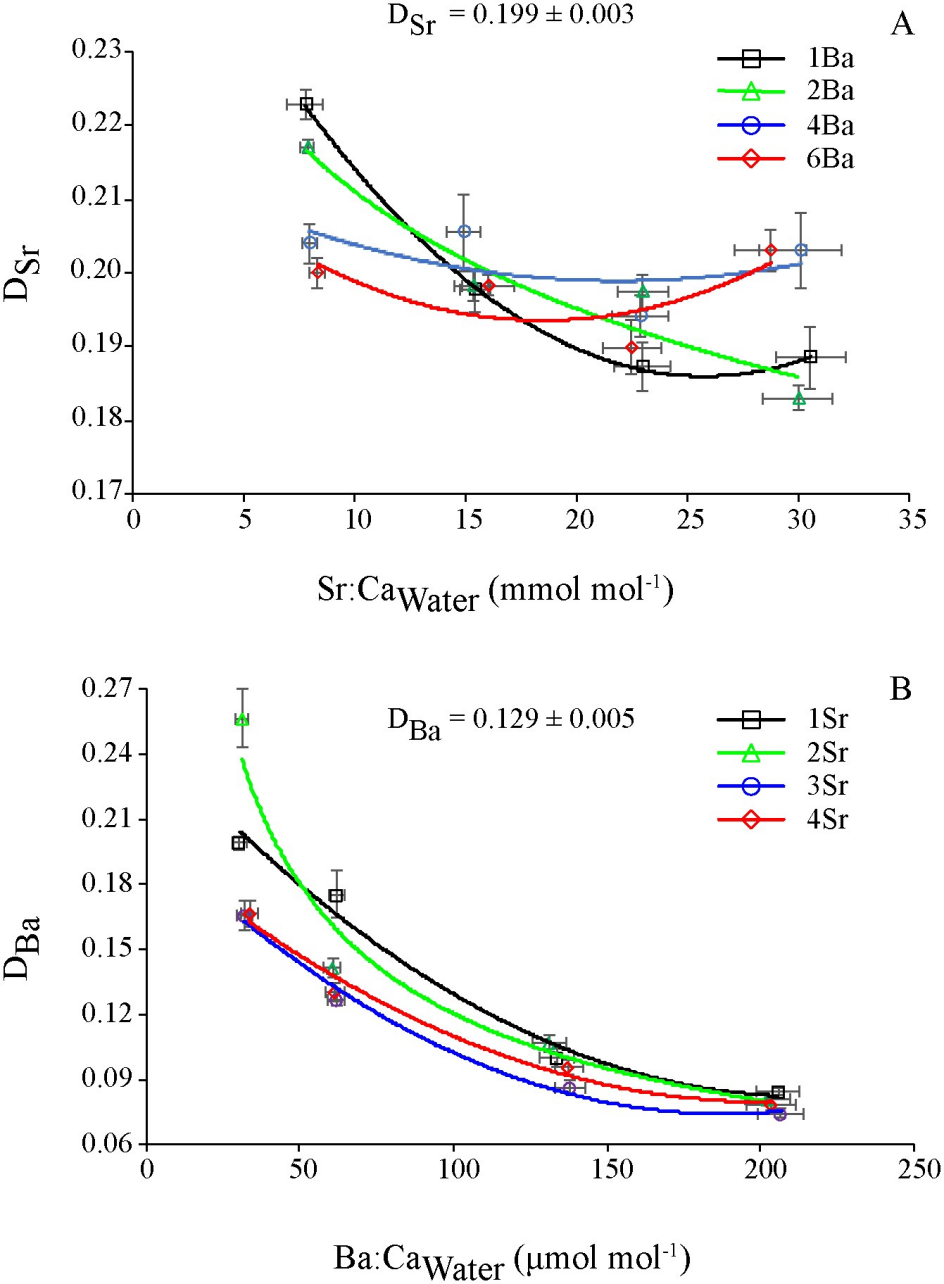
Plots of mean D_Me_ vs. ambient Me:Ca_Water_ for Sr (A) and Ba (B). The lines of the best trends represent the trends at different elemental concentrations. Data represent the mean values of D_Me_ or Me:Ca_Water_ for each treatment.

D_Sr_ values were 0.19–0.22, 0.18–0.22, 0.19–0.20 and 0.19–0.20 among the Sr concentrations at 1Ba, 2Ba, 4Ba and 6Ba concentrations, respectively (Fig 5A). D_Sr_ tended to decrease as Sr:Ca_Water_ increased at low concentrations(1Ba and 2Ba), but remained relatively constant among Sr concentrations at high concentrations (4Ba and 6Ba).

D_Ba_ values were 0.08–0.19, 0.08–0.25, 0.07–0.17 and 0.08–0.17 among the Ba concentrations at 1Sr, 2Sr, 3Sr and 4Sr concentrations, respectively (Fig 5B). At each Sr concentration, D_Ba_ tended to decrease as Ba:Ca_Water_ increased.

## Discussion

### Interactive effects of water elements on otolith elemental incorporation

One of the fundamental assumptions for applying otolith microchemistry to fish ecology studies is that variations in otolith elemental incorporation primarily reflect changes in ambient water chemistry [1, 2, 4, 12]. A number of previous experimental studies documented the relationship between otolith elemental incorporation and elements in ambient water [14, 27]. Reported Me:Ca_Otolith_ values differ among studies depending on the fish species, water elemental concentrations and experimental conditions (S1 Table). Some studies showed that positive relationships occurred between the elemental concentrations in otoliths and water [2, 4, 14, 27]. However, other studies found that the otolith Sr:Ca ratio may not be a reliable indicator of the environment because otolith elemental incorporation can be regulated or influenced by many other factors, especially physiological factors. [12, 16]. In our study, both Sr:Ca_Water_ and Ba:Ca_Water_ across treatments fell within the ranges (3.34–150 mmol mol^-1^ for Sr and 3.3–230 μmol mol^-1^ for Ba) measured in previous experimental studies except for the high Ba value (4610–6320 μmol mol^-1^) observed in Collingsworth et al. [23] (S1 Table). Although otolith elemental concentrations differed across treatments and among individual otoliths within each treatment in some cases, no elemental data were discarded during data analyses because the CV within each treatment was relatively low (2.9–8.1% for Sr and 5.8–10.5% for Ba). The average Sr:Ca_Otolith_ and Ba:Ca_Otolith_ of each treatment fell within the ranges (*c*.0.59–8.00 mmol mol^-1^ for Sr and *c*.0.53–15.20 μmol mol^-1^ for Ba; S1 Table) reported in other marine fish species. Both Sr:Ca_Otolith_ and Ba:Ca_Otolith_ increased with increasing water elemental concentration, thus validating the abovementioned fundamental assumption for otolith microchemistry studies.

Of the confounding environmental variables, water temperature and salinity are often tested to examine their interactions with water elemental concentrations and their impacts on otolith elemental incorporation in experimental studies [11, 25–30]. In most of the studies, a single specific trace element was addressed independently. However, the influence of certain processes, such as competition and facilitation among multiple elements on otolith microchemistry has not been adequately tested or evaluated, although this “elemental effect” has been reported in some fish [15, 23]. Of the few studies that addressed this issue, de Vries et al. [15] found that increasing Sr water concentrations could facilitate the uptake of Ba into the otoliths of juvenile black bream reared in high salinity brackish water, although not in individuals reared in seawater [15]. Conversely, Ba neither inhibited nor facilitated Sr uptake in black bream reared either in brackish or seawater. In freshwater yellow perch juveniles, a positive relationship between D_Ba_ and Sr:Ca_Otolith_ was detected [23].

Our study attempted to keep the experimental environment consistent across treatments, assuming that water elemental concentration was the main variable that affected otolith elemental incorporation. Ba:Ca_Water_ neither inhibited nor facilitated Sr:Ca_Otolith_ in flounder, agreeing with the results in the literature. However, Ba:Ca_Otolith_ showed an overall tendency of decreasing with increasing Sr:Ca_Water_, particularly at low Ba water concentrations. High Sr levels might negatively affect the uptake of Ba into otoliths, although its effect might not be as significant as that of the Ba concentration in the environment. This finding was further supported by the negative relationship that was detected between Sr:Ca_Water_ and Ba:Ca_Otolith_ at the same Ba water concentration. This result appeared to be inconsistent with that of de Vries et al. [15], where they noted that ions are competing for space in the otolith matrix. Nonetheless, this idea was derived from an experiment in which fish were reared in freshwater or high salinity brackish water with different ambient elemental concentrations and has not yet been reported in fish reared in seawater. The process by which Sr water concentration affects Ba uptake into otoliths could be regulated by the salinity and elemental baselines in the environment, as revealed by the different “elemental effects” occurring in black bream reared in brackish water and seawater (S1 Table). The facilitation of Ba incorporation into otoliths by Sr might be exclusive to freshwater or brackish water with high Sr:Ca_Water_ or Ba:Ca_Water_ ratios. The differences in experimental conditions among studies might also confound otolith elemental uptake, resulting in inconsistent results.

### Efficiency of otolith elemental incorporation from ambient water elements

The partition coefficient (D_Me_) is a predictable indicator for assessing the efficiency of elemental incorporation into otoliths from ambient water. D_Sr_ appears to have a relatively consistent range in experimental studies, even though the studies differed in experimental design (S1 Table). In some cases, certain factors may affect the efficiency of otolith elemental incorporation. For example, D_Sr_ was positively temperature-dependent in freshwater yellow perch [23] or was negatively concentration-dependent on Sr in black bream in seawater, although remained relatively constant in brackish water [15]. Additionally, with the exception of a high value (0.53) in black bream in a field study [18], D_Sr_ was generally larger in fish reared in freshwater or brackish water than in seawater [13, 15] (S1 Table). This result was well exemplified by the experimental findings in black bream reared in brackish water (0.463) and seawater (0.130–0.287) [15, 18, 27]. The difference in D_Sr_ among fish reared in different aquatic environments could be caused by the mechanisms by which fish regulate otolith elemental incorporation or was likely related to the Sr concentrations and salinities in the environments. In our study, D_Sr_ ranged from 0.18 to 0.22, which is comparable to the values (0.13–0.30) reported in marine fish in experimental studies (S1 Table). Contrary to Sr:Ca_Otolith_, which was positively concentration dependent on Sr:Ca_Water_, D_Sr_ showed an overall tendency of decreasing with increasing Sr:Ca_Water_ in low Ba concentrations and fluctuated within a small range at high Ba concentrations. Similar trends of Sr incorporation into otoliths were also observed in other fish species reared in seawater [15]. It was reasonably assumed that the rate of Sr:Ca_Otolith_ increase might be low compared to that of Sr:Ca_Water_ over the course of the experiment, resulting in a negative relationship between D_Sr_ and Sr:Ca_Water_. At each Sr concentration, D_Sr_ was relatively constant in all Ba concentrations, indicating that Ba was unlikely to play a significant role in regulating the efficiency of Sr incorporation into otoliths.

The D_Ba_ ranged from 0.08 to 0.25, which is comparable to the values in other fish species (0.058–0.430) except for yellow perch (*c*. 0.008, high Ba:Ca_Water_ baseline; S1 Table) [23]. Overall, D_Ba_ was lower than D_Sr_ across treatments, suggesting that Sr was likely to be incorporated into otoliths more efficiently than Ba. D_Ba_ showed an apparent trend of decreasing with increasing Ba:Ca_Water_ at all Sr concentrations. This trend indicated that the efficiency of Ba incorporation into otoliths differed with ambient Ba concentrations irrespective of Sr concentration. The decreasing trend of D_Ba_ with increasing Ba water concentration did not vary much among species and studies [14, 15, 27]. The increasing rate of Ba incorporation into otoliths was low compared to the increase in Ba water concentration, partly accounting for the negative relationship between D_Ba_ and Ba:Ca_Water_. However, the trend in which D_Me_ decreased with increasing Me:Ca_Water_ for both Sr and Ba exhibited a nonlinear relationship. As the water elemental concentration increases, ions incorporated into otoliths may become close to saturation in the otolith crystal matrix, slowing the efficiency of otolith elemental incorporation. D_Me_ may stabilize after a dynamic equilibrium is reached in ions among the ambient water, interface and otolith matrix at a certain water elemental concentration [15]. Despite the fact that the abovementioned trends for both Sr and Ba were observed in various studies, the D_Me_ values for an element may fluctuate within a certain range among fish species and studies since environmental and physiological variables are likely to participate in regulating the activities and behaviors of the elements during otolith elemental incorporation.

## Conclusions

The incorporation of Sr and Ba in otoliths of juvenile flounder was positively dependent on the concentrations of the elements in water. Overall, Sr was more efficiently incorporated into otoliths than was Ba. The partition efficiencies of Sr and Ba were negatively correlated with the water elemental concentrations, suggesting that the efficiency of otolith elemental incorporation decreased with increasing elemental concentration. Increasing Sr water concentration appeared to negatively affect the uptake of Ba into otoliths. However, the Ba concentration in water did not affect Sr otolith uptake, agreeing with the findings for other fish. Thus, the impacts of interactions of multiple elements in ambient water on otolith elemental incorporation must be addressed when applying otolith microchemistry to field studies of fish ecology.

## Supporting information

S1 Table Summary of the results of the relationships between otolith elemental incorporation and water elemental concentration for Sr and Ba in the literature*. (DOCX)
