# Supplementary material for: Interactive effects of strontium and barium water concentration on otolith incorporation in juvenile flounder Paralichthys olivaceus
Source: PLoS One. 2019 Jun 14;14(6):e0218446. doi: 10.1371/journal.pone.0218446 (PMC6568404; doi:10.1371/journal.pone.0218446)
Supplement: S1 Table — (DOCX) [file pone.0218446.s001.docx]

**S1 Table. Summary of the results of the relationships between otolith elemental incorporation and water elemental concentration for Sr and Ba in the literature***

| Fish | | Study designing | | | Otolith elemental incorporation | | | | Sources |
| --- | --- | --- | --- | --- | --- | --- | --- | --- | --- |
| Species | Life stage | Ambient | Type | Sr:Ca_Water_ (mmol mol^-1^)  Ba:Ca_Water_ (μmol mol^-1^) | Sr:Ca_Otolith_  (mmol mol^-1^) | D_Sr_ | Ba:Ca_Otolith_  (µmol mol^-1^) | D_Ba_ |  |
| Spot  *Leiostomus xanthurus* | Juvenile | Sea water | Exp | 20°C and 25°C  Sr: 1x-1.8x; 12-23 Ba: 1x-10x; 23-230 | 1.85-6.77  (20 and 25°C) | 0.182 (20°C)  0.205 (25°C) | 1.7-15.2  (20 and 25°C) | 0.060  (20 and 25°C) | Bath et al. (2000) |
| Yellow perch *Perca flavescens* | Juvenile | Freshwater | Exp | 10°C, 15°C and 20°C  Sr: 3.34-15.87 Ba: 4900-6320 | c.0.70-1.50 (10°C) c.0.80-1.70 (15°C) c.0.70-2.50 (20°C) | c.0.21 | c.2.0-8.0 (10°C)  c.2.0-7.0 (15°C)  c.1.0-8.0 (20°C) | c.0.008 | Collingsworth et al. (2010) |
| Black bream *Acanthopagrus butcheri* | Juvenile | Euryhaline | Exp | Salinity 5 and 32 Sr: 1x-3x; c.10-150 (S5); c.7-30 (S32) Ba: 1x-3x; c.20-130 (S5); c.5-25 (S32) | c.5-70 (S5) c.3-10 (S32) | 0.463 (S5) 0.287(S32) | c.1.5-8.0 (S5) c.1.0-2.0 (S32) | 0.058 (S5) 0.136 (S32) | de Vries et al. (2005) |
|  | Juvenile | Sea water | Exp | Sr: 1x-16x, 10-125  Ba: 1x-16x, 5-30 | c.2-12 | 0.13 | c. 1.0-2.5 | 0.099 | Elsdon and Gillanders (2003) |
|  | Juvenile | Euryhaline | Exp | 17°C and 26°C  Salinity 5 and 32 Sr: 1x and 8x; c.5-150 Ba: 1x and 8x; c.5-280 | c.4.0-43.0 (17°C) c.3.0-58.0 (26°C) c.4.0-58.0 (S5) c.3.0-8.0 (S32) | NA | 2.0-10.0 (17°C) 3.0-16.0 (26°C) 2.0-16.0 (S5) 3.0-10.0 (S32) | NA | Elsdon and Gillanders (2004) |
|  | Juvenile/Adult | Sea water | Field work | Sr: c.2-23 Ba: c.10-210 | c.2.8-5.1 | 0.52 | c.1.0-15.0 | 0.260 | Elsdon and Gillanders (2005) |
| Black rockfish *Sebastes melanops* | Juvenile | Sea water | Exp | 8 and 13°C  Ba: 1x-12x; 18-206 | NA | NA | 1.67-10.38 | 0.048 (8°C) 0.061 (13°C) | Miller (2009) |
| European bass  *Dicentrarchus labrax* | Juvenile | Euryhaline | Exp | 21 and 25°C  Salinity 10,20 and 32 Sr: 1x-10x; c.10-100 Ba: 1x-10x; c.17-170 | c.2.0-28.0 (21°C) c.3.0-30.0 (25°C) c.2.0-30.0 (S10) c.2.0-13.0 (S20) c.2.0-8.0 (S30) | 0.22-0.36 | c.1.0-8.0 (21°C) c.1.0-9.0 (25°C) c.1.0-9.0 (S10) c.1.0-7.0 (S20) c.2.0-6.0 (S30) | 0.040-0.1234 | Reis-Santos et al. (2013) |
| Atlantic silverside *Menidia menidia* | Larvae | Sea water | Exp | T: 15, 21 and 27°C Sr: c. 8.7-8.8 Ba: 7.5-8.6 | c.2.0-2.1 (15°C) c.2.0-2.1 (21°C) c.2.5-2.6 (27°C) | c.0.20-0.25 (15 and 21°C) c.0.30 (27°C) | c.1.8-2.4 (15°C) c.2.1-2.6 (21°C) c.2.1-3.2 (27°C) | c.0.18-0.32 (15°C) c.0.16-0.18 (21°C) c.0.26-0.43 (27°C) | Clarke et al. (2011) |
| Flounder  *Paralichthys olivaceus* | Juvenile | Sea water | Exp | Sr: 1x-4x; 7.77-30.54 Ba: 1x-6x; 30.5-206.5 | 1.62-6.11 | 0.18-0.22 | 5.32-16.44 | 0.08-0.25 | Present study |
| Westslope cutthroat trout *Oncorhyncus clarki lewisi* | Juvenile/Adult | Freshwater | Field work | Sr: 0.54-6.14  Ba: 290-5110 | 0.12-1.61 | 0.40 | 30.0-190.0 | 0.04 | Wells et al. (2003) |
| Australasian snapper *Pagrus auratus* | Juvenile | Sea water | Field work | Sr: 7.42-7.52 Ba: 5.84-11.94 | 0.59-0.60 | 0.08 | c.2.51-5.13 | 0.43 | Hamer and Jenkis (2007) |
| Sand flathead  *Platycephalus bassensis* | Juvenile | Sea water | Field work | Sr: 7.42-7.52 Ba: 5.84-11.94 | 0.74-0.75 | 0.10 | c.0.53-1.07 | 0.09 | Hamer and Jenkis (2007) |
| Common sole *Solea solea* | Juvenile | Sea water | Field work | Sr: c.6.5-9.0 Ba: c.10-90 | c.1.9-3.2 | 0.27-0.36 | c.0.9-7.2 | 0.07-0.12 | Tanner et al. (2013) |

*values with c. were estimated from the figures in the literature, and the others are the originally reported values.
